# Physiological Relations of Gular Vocalization in Grouse

**Published:** 1888-04

**Authors:** G. Archie Stockwell

**Affiliations:** Port Huron, Mich.


### Art. X.—PHYSIOLOGICAL RELATIONS OF GULAR VOCALIZATION IN GROUSE.

BY G. ARCHIE STOCKWELL, M. D., F. Z. S.
*Port Huron, Mich.*

Spring, with its wealth of verdure and bloom, brings a flood of melody to orchard, meadow and forest. It is love-time among the birds.

As the season approaches, the tendency of all feathered males, is toward ostentation, and display of the charms that are inherent by reason of race and sex. Those of more sober dress and modest mien, woo their mistresses with soft and fervid notes; others by the aid of gorgeous apparel; some again, denied gifts of voice and fine plumage, resort to gallantry like knights of old, and seek to display to best advantage, certain specific adornments that, may or may not, endow the possessor with a supplemental and spurious vocality. In each and every instance it is the old and self-same story—the desire to implant the seeds of love in the female breast; and the old Moorish sage, Bonabbon, taught better than he knew when he declared this to be

"the cause of half the ills of wretched mortality," since among the higher orders of animality, consummation of the tender passion is rarely effected without more or less of heart-burnings and jealous strifes.

Above all, the *gallinaceæ* are most conspicuous for the arrogance and pride that is born of polygamous relations, and lack of true domesticity. The males are the epitome of everything that is lustful, arbitrary and selfish ; and their mistresses, little aside from mere wantons, are swayed by passion and caprice, and their best allegiance is obtained by exercise of force. This is constantly seen among the in habitants of the barnyard, where the male *gallus* in conscious pride of Oriental ancestry, yet awakens the *poulardes* at dawn to pay court, and view exhibitions of parade and strut calculated to inflame their servile passions; or in the turkey-cock, with a lineage derived from Mexican jungles and coverts, who summons the seraglio with loud and sonorous *gobble* to admire his turgid wattles, buzzing wings, and a metallic plumage flaunted in the sun.

Among the *tetraonidæ* may be observed two peculiar performances tending to the same end, and that with propriety, are designated respectively, *solitary* and *gregarious* courtship. Neither is essentially specific, but the outcome rather, of habits of life enforced by surroundings —the latter perhaps from a demand for mutual protection from foes, including climate. Grouse of the forests and mountains, better guarded by reason of plumage in atune with the salient features of their habitat, and abundantly provided with shelter, are invariably solitary wooers. *Per contra*, they of the prairies and more sparsely wooded districts (the ptarmigans for obvious reasons excepted), present the reverse habit The blue or dusky grouse, *tetrao obscurus*, as a denizen of the woodlands to the west of the Rocky Mountains, perches in lonesome grandeur upon some branch of fir or spruce, flaunts his wattles, and causes the forest aisles to reverberate with echoes that lure the pretendedly coy, but only too-eagerly lascivious hens ; but when found upon the prairies, or in the stunted groves of the hither slope of the great continent-dividing range, he is uncompromisingly gregarious in his courtships, and mocks the "dancings" and toolings of the sharp-tails and prairie hens (*t. phasianellus* and *t. cupido**). It may be worthy of remark also, that once the sage cock, or "cock of the plains" (*t. urophasianus*—the rival of the Old World capercalzie *t. urogallus*), and a whilom tree-percher of the forests of the upper Canadian and Arkansas, was wont to simulate his dusky relative of the Pacific Coast; but driven by circumstances to the open plains, the males of the species now gather in groups, and strut and vie one with another, while bidding for the favors of the gentler sex. This is in some sense also, true of *t. phasianellus*, and that, too, within the memory of an existing generation, with whom the gular development is coincident with the change of habits.†

Solitary wooers, owing to peculiarities of love vocalization, obtain the aptly, though vulgarly, designated pseudonym of "drummers," and aside from the *Scolopacidæ*, are represented by but two species, *tetrao canadensis* and *t.* (*bonasa*) *umbellus*, or spruce and ruffed grouse. Gregarious wooers in like manner are specified as "boomers," a popular appellation in some sense descriptive of the love and song of their class, and these last include four species: *tetrao cupido*, *t. obscurus*, *t. urophasianus* and *t. phasianellus*—pinnated, dusky, sage and sharp-tailed grouse.

"Boomers," in exaggerated form, exhibit a supplemental gular apparatus, an ornamentation that is now known to be functionally and physiologically allied to the breeding habit. Markedly the prerogatives of the male sex, and merely adjunctive, they are no way essential, save as aids wherewith to secure the favors of the opposite sex. Loss or lack of development jeopardizes neither virility, or the

* For obvious reasons the nomenclature of the Br. Museum is preferred to the fanciful, arbitrary, and finical subdivisions that of late have secured adoption in the United States, causing the genera to rival the species in number.

† So true is this, that the sharp-tailed grouse is generally held and described as wanting in gular ornamentation, especially in Michigan, and elsewhere where they have not been compelled to abandon their forest home. In the Northwest they have been driven to the prairies, and, in some instances, to mingle and breed with *t. cupido*, and here the gular development is already becoming marked.

welfare of the general economy, and it is not uncommon, especially in old birds, to find them obliterated by disease—by the ravages of larvæ and parasitical forms of life that have found refuge therein, or rendered useless as the sequel of wounds inflicted by jealous rivals; caponizing arrests their growth in young birds, causing to shrivel and absorb, and the latter is also equally true of adults, though slower in degree.

Strictly speaking, however, these appendages are by no means singular, but obtain of greater or less development in all grouse, females as well as males; but with the former, and likewise with the "drummers," they are merely rudimentary, and unavailable save as the result of freak or accident. Hens, that exhibit prominent gulars, usually obtain the ornamentation at the expense of other portions of the reproductive apparatus, for as they do not ovulate, they are incapable of fecundation, and oftentimes the victims of perverted or reversed sexual appetite. The anatomical changes usually observed under such circumstances are, imperfect development, displaced or diseased ovaries, or perhaps, utter lack of these organs. A bird recently dissected, supposed to be a male because in possession of an unusually fine pair of gulars, gave no evidence of intromittent organ, or of testes or ovaries, and was relegated to the opposite sex owing to the relations of the cloaca, and presence of a rudimentary oviduct. Hermaphrodism, so frequently claimed for "booming" hens with opposite sex tendencies, so far as I am aware, has never been satisfactorily substantiated; and, as is well known, true bi-sexualism, or more than slight relations thereto, is one of the rarest of phenomena observed among higher vertebrates.

Gular ornaments in grouse, are but the analogue of *wattles* in domestic fowl and other *gallinaceæ;* their intent and purpose are practically the same, the differences being chiefly of form and arrangement. Of spherical or ovate outline, they appear as pouches or sacs attached to either side of the anterior portion of the upper neck near the articulation of the lower maxillary—the situation being nearly allied to that of the thyroid gland in higher orders. When quiescent they are discernible only as mere patches of loose, corrugated integument, seemingly too ill-nourished for the support of pennate growth, and partially or wholly concealed by the luxuriant plumage of the hackles. Under the stimulus of sexual ardor, however, all this is changed: the wrinkled skin, that before resembled common integument, swells, dilates and distends, assuming a smooth, tense contour, taking deep red-yellow or orange hues, and well justifies the comparison commonly made to fruit of the *citrus*. Now they have a capacity of from three to eight, or even ten and twelve fluid ounces, according to species, and in some rare instances (as in *t. urophasianus*) of a full pint; and instead of separate organs, they assume rather the form of two lobes of the same structure, which in some instances becomes an absolute fact, the division being but partial and the separation by extremely thin membrane. These changes are not brought about by inflation and distension with air, as generally surmised, so much as by erethrism developed through determination blood to the layer vascular tissue that exists between the membranous lining and outer integument, and, indeed, it is somewhat questionable if air plays any part in the functions of these organs, other than as may be required for vocalization.

The membrane that sheaths the interior of the gulars, is a proliferation of the mucous lining of the pharynx and mouth, through a brief common duct. Each sac sometimes developes its own duct, and sometimes two ducts have a common orifice divided into two apertures by means of a septum. None of these features of duct formation are at all constant, however, even in the same species, since three birds selected at random may exhibit as many variations. In all, the external orifice (or orifices) will be found in the midst of the sub-lingual space, just anterior to the larynx, and between the latter and the deep sub-mandibular and cutaneous tissues. By putting the bill upon the stretch as in the act of gaping, and lifting the tongue from its resting place between the *rami* of the lower mandible, the aperture is revealed; or, if desirable to further facilitate exploration, stretch the neck, tightly compressing it behind the gulars, then lift or push the tongue back a little, (observe how it fits the groove with all the precision of a valve and effectually closes the orifice) and inflate by blowing in the mouth, subsequently allowing the air to slowly escape from the sacs.

Beneath the common integument, and external to an aponeurotic capsule surrounding the vascular and erectile layer, will be observed traces of connective tissue, with here and there stray or isolated fibres of *panniculus carnosus.* Passing on to the erectile tissue, we find it consists chiefly of a dense net-work, formed by interlaced nerve fibrils, and anastamosed arterioles and venules, that duly account for the erethrism on occasion obtaining; the nerve supply is largely traceable to the *ganglion cervicale primum,* the fibrils being intricately interwoven with a less number indirectly derived from the *ganglion gasserius.*

During the continuance of erotomania, and when the love-song is in progress, the sacs are synchronously inflated and exhausted, producing deep rolling or booming notes of peculiar measure and rhythm, more or less puzzling to the listener because of their uncertain and ventriloqual accentuation. With care, two separate and distinct sounds may be discerned; one interrupted with some pretense to regularity, and purely gular in that it subsides coincidentally with erethrism and inflation; the other prolonged, continuous and subjective, like the *drone* of the bag-pipe. This last, pure and simple, the music of the "*drummer,*" is derived from the resistance of air to the swiftly vibrated pinions, every feather of which is rigidly segregated, the fibrils meantime erected and fixed, securing as it were, a pennate form of Æolian harp.

At such time the performer appears as if in the agonies of suffering from physical distortion, though quite the reverse is truth, since he really experiences the delights of partial ecstasia induced by general (sexual) nerve orgasm. The neck is recurved until head and stiffened hackles are made to greet the expanded anteverted cauda; the mandibles, wide agape, are perpendicularly lifted to bring the oral space as nearly in line with pharynx and larynx as the anatomy of the parts will permit, whereby the sacs are thrust prominently forward and the folds of the gular duct obliterated, admitting free entrance and exit to air; meantime the tongue vibrates with inconceivable swiftness, affording ground for the presumption it is the sole agent of inflection and modulation, and that the rolling, booming character of the gular notes, like those of the pinions, is due to the sharpness and rapidity with which they are accented and interrupted.

Inflation of the sacs is apparently accomplished by a *catch* in inspiration, whereby is had a lifting and partial interposition of the hyoidean apparatus, the bill being closed for the purpose; it is in some measure the reverse of the respiratory act as observed in turtles and kindred reptiles. Exhaustion, presumably, is the sequel of muscular contraction in which the fibres of the *panniculus carnosus*, before noted, probably play an important part, aided by neurotic laxity, and relaxation of arterioles. Expansion and inflation of the body, so generally claimed as an attendant upon "booming," is anatomically and physiologically impossible, save as such obtain to all birds; and the deception is largely due to the general upset condition of the plumage from cauda to vertex, that convey to the beholder the ludicrous suggestion of an attempt of the creature to turn itself wrong-side out; also it arises in part from contractions of the muscles of the upper thorax and neck, that are observed to occur coincidently with the vibratory movements of the pinions, hackles, and exhaustion of the gulars.

The most elaborate and complex form of gular apparatus undoubtedly is that of *meleagris gallipavo*—the wild turkey—and its domesticated congener; this though single, is yet appartmental by virtue of being repeatedly flexed upon itself. *Per contra*, the least complex, pertains to the common bustard of Europe, *otis tardæ*, being merely a simple development, and a sac of such ample dimensions it was long held to subserve a purpose like that of the fifth, or recticulated portion, of the compound stomach of certain higher *ruminantia*—the camel, for example—an error, I believe, that devolved upon the late Doctor Frank Buckland to explode, who was so fortunate as to detect a cock bustard in the very act of chanting his wooing song. To this hour it is no uncommon feature of popular works of natural history to find it described as: "An admirable provision of nature to subvert the tortures of thirst;" "A magazine against drouth capable of conveying *seven (?) quarts* of water"; "filled on proper occasions to supply the demands of the female while engaged upon the nest in the duties of incubation"; and to "quench the thirst of the young brood before they are sufficiently developed to employ their pinions," etc.* And this in the face of the fact the bustard was known to shun water, and that the pinnated grouse and other gular songsters, as *feræ naturæ*, are inhabitants of dry, and often arid uplands, sparing in the use of fluids, and accustomed to quench thirst by means of dew and other moisture gathered drop by drop, from off the ground and herbage.

Singly pouched, or wattled, birds are less capable of vocality, as a rule, than those possessed of double or compound sacs, and the range and volume of sound is more restricted; but single wattles always exhibit a tendency toward the bi-gular form, even if in nothing more than a strongly outlined and thickened *raphé*. An African form of bustard is reported as possessing a single pouch divided at its most depended extremity, and more anteriorly than posteriorly, by a semi-lunar septum; but this, if true, in no way adds to our stock of physiological knowledge; it is of interest chiefly to the evolutionist as one of many links in a series. Also, it has twice been my privilege to see double wattles in the meleagrine fowl, though the superflous ornament, in one instance at least, was had at the expense of vocality.

The love jousts or "dances" to accept the popular vernacular of gregarious wooers, are well known through the

---

* *Vide* Gould, Buffon, Gooderich, Goldsmith and Wood.

medium of Wilson, Audubon, Giraud and others, hence require nothing at my pen in this connection. Most writers, however, surmise them to be mere mock parades and tournaments gotten up for the delectation of the weaker sex; and though this is in some sense and measure true, it is very far from being the rule. I have repeatedly discovered wounded, maimed, dead and dying birds, upon the field of battle, and once picked up a cock minus an eye, plus a broken wing, with head nearly severed from the body, manifestly the work of the bills of rival opponents. Doubtless the fatal strokes were directed at the gulars, these being the invariable objects of wrath; and here also may be the key to the instinct which secures mortal aversion to *red* on the part of nearly, or quite all gallinaceous fowls.

The brunt of the conflict is always sustained by the younger birds, who, when earnestly engaged, are slyly deserted by their older and more astute rivals; evidently the latter prefer the court of Venus to the camp of Mars; and by this bit of *finesse*, the latter secure the majority of hens, who are supposed, always to be concealed witnesses of these performances. A dispute between two elderly cocks, when each has a following of hens at his tail, is truly a battle royal, though lacking in the music and martial display peculiar to youngsters. In such case it is rare that either get off without punctured gulars; and the victor invariably merges the harem of his rival into the ranks of his own following. The salacity of both cocks and hens, moreover, is surprising beyond computation, and may account in some measure for the fact, all *gallinaceœ* exhibit more or less of epileptic neurosthenia. It would be interesting to know more of the psychic relations of the sexual function in these as well as other creatures.

